# The role of coronary lipid burden in immediate atherosclerotic non-culprit plaque regression following acute coronary syndrome^☆^: A post-hoc analysis of the FITTER trial

**DOI:** 10.1016/j.ajpc.2026.101772

**Published:** 2026-07-27

**Authors:** Jonathan Los, Frans B. Mensink, Rohit M. Oemrawsingh, Alexander J.J. Ijsselmuiden, Martijn Meuwissen, Jin M. Cheng, Himanshu Rai, Tim J.F. ten Cate, Cyril Camaro, Peter Damman, Lokien X. van Nunen, Aukelien C. Dimitriu-Leen, Marleen H. van Wely, Aysun Cetinyurek-Yavuz, Robert A. Byrne, Niels P. Riksen, Saloua El Messaoudi, Jan H. Cornel, Niels van Royen, Robert-Jan M. van Geuns

**Affiliations:** a Department of Cardiology, Radboud University Medical Center, Nijmegen, the Netherlands; b Department of Cardiology, Albert Schweitzer Hospital, Dordrecht, the Netherlands; c Department of Cardiology, Amphia Hospital, Breda, the Netherlands; d Department of Cardiology, Maastricht University Medical Center, the Netherlands; e School of Pharmacy and Biomolecular Sciences, RCSI University of Medicine and Health Sciences, Dublin, Ireland; f Cardiovascular Research Institute (CVRI) Dublin and Department of Cardiology, Mater Private Network, Dublin, Ireland; g Department of IQ Health, Radboud University Medical Center, Nijmegen, the Netherlands; h Department of Internal Medicine, Radboud University Medical Center, Nijmegen, the Netherlands; i Department of Cardiology, Northwest Clinics, Alkmaar, the Netherlands; j Dutch Network for Cardiovascular Research (WCN), Utrecht, the Netherlands

**Keywords:** Intravascular ultrasound (IVUS), Near-infrared spectroscopy (NIRS), Vulnerable lesions, Lipid-rich plaque

## Abbreviations and acronyms

ACS: Acute coronary syndrome
EEM_area_: External elastic membrane area
FFR: Fractional flow reserve
FITTER: Functional Improvement of non-infarcT relaTed coronary artery stenosis by Extensive low-density lipoprotein cholesterol Reduction with a proprotein convertase subtilisin/kexin type 9 antibody
IVUS: Intravascular ultrasound
LDL-C: Low-density lipoprotein cholesterol
LCBI: Lipid core burden index
MaxLCBI_4mm_: Maximum lipid core burden index within any 4-mm segment
MLA: Minimum lumen area
NIRS: Near-infrared spectroscopy
PAV: Percent atheroma volume
PB: Plaque burden
PCI: Percutaneous coronary intervention
TAV: Total atheroma volume

## 1 Introduction

Lipid-lowering therapy is essential for reducing atheroma volume and decreasing coronary lipid burden [1]. However, the relationship between changes in coronary lipid content and atheroma dimensions remains poorly understood. On the one hand, coronary segments displaying high lipid content may respond more readily to lipid-lowering therapy and demonstrate increased atheroma size reduction. Conversely, the vulnerability represented by lipid-rich plaque may contribute to atheroma progression. The FITTER trial was designed to assess the immediate effect of very-high intensity lipid-lowering therapy on relevant non-culprit lesions in patients presenting with acute coronary syndrome (ACS) [2]. In this post-hoc analysis, we evaluated the impact of coronary lipid burden on early changes in atheroma volume.

## 2 Methods

### 2.1 Study design and population

The FITTER trial (NCT04141579, enrollment November 2020 - August 2023) was a multicenter, double-blind, placebo-controlled trial that randomly allocated 150 patients with ACS and multivessel disease to receive evolocumab or placebo in addition to high-intensity statin therapy for 12 weeks [2]. Ethical approval was given by the local ethical review board and all participants provided written informed consent. Patients were included if successful percutaneous coronary intervention (PCI) of the culprit vessel was performed, and if they demonstrated at least one relevant non-culprit stenosis (fractional flow reserve [FFR] measurement of 0.67 to 0.85) in a non-infarct-related artery amenable for PCI. After 12 weeks of therapy, coronary angiography was repeated. In 86 patients, additional serial intravascular ultrasound-near-infrared spectroscopy (IVUS-NIRS) imaging was performed as well. Regarding the main endpoints, the addition of evolocumab had no significant short-term effect on intracoronary physiology, coronary lipid burden, or atheroma volume compared with high-intensity statin therapy alone [2,3]. Therefore, to investigate the dynamic interplay between coronary lipid burden and atheroma volume, treatment groups were pooled for the present study.

### 2.2 IVUS-NIRS imaging acquisition and imaging outcome measures

Details regarding FFR measurements, IVUS-NIRS imaging acquisition, imaging outcome measures, and data analysis were previously described [2]. For each imaged vessel, the percentage atheroma volume (PAV), total atheroma volume (TAV), external elastic membrane area (EEM_area_), maximum plaque burden (PB), minimum lumen area (MLA), and the 4-mm maximum lipid core burden index (maxLCBI_4mm_) were obtained. The maxLCBI_4mm_ represents NIRS-derived coronary lipid burden, with values ≥325 indicating lipid-rich plaque associated with an increased risk of recurrent cardiovascular events [4]. To evaluate the regional effect of lipid burden on atheroma expansion, the imaged coronary arteries were divided into 20-mm segments (20 frames) beginning at the proximal end of each matched vessel, from which identical plaque volume parameters were derived (Fig. 1B). Distal remnant segments of less than 4 frames, were merged with the preceding segment to enable calculation of segment-level maxLCBI_4mm_ values. Vessel- and segment-level TAV and EEM_area_ were normalized to the median number of frames across all vessels and segments, respectively.

**Fig. 1:**
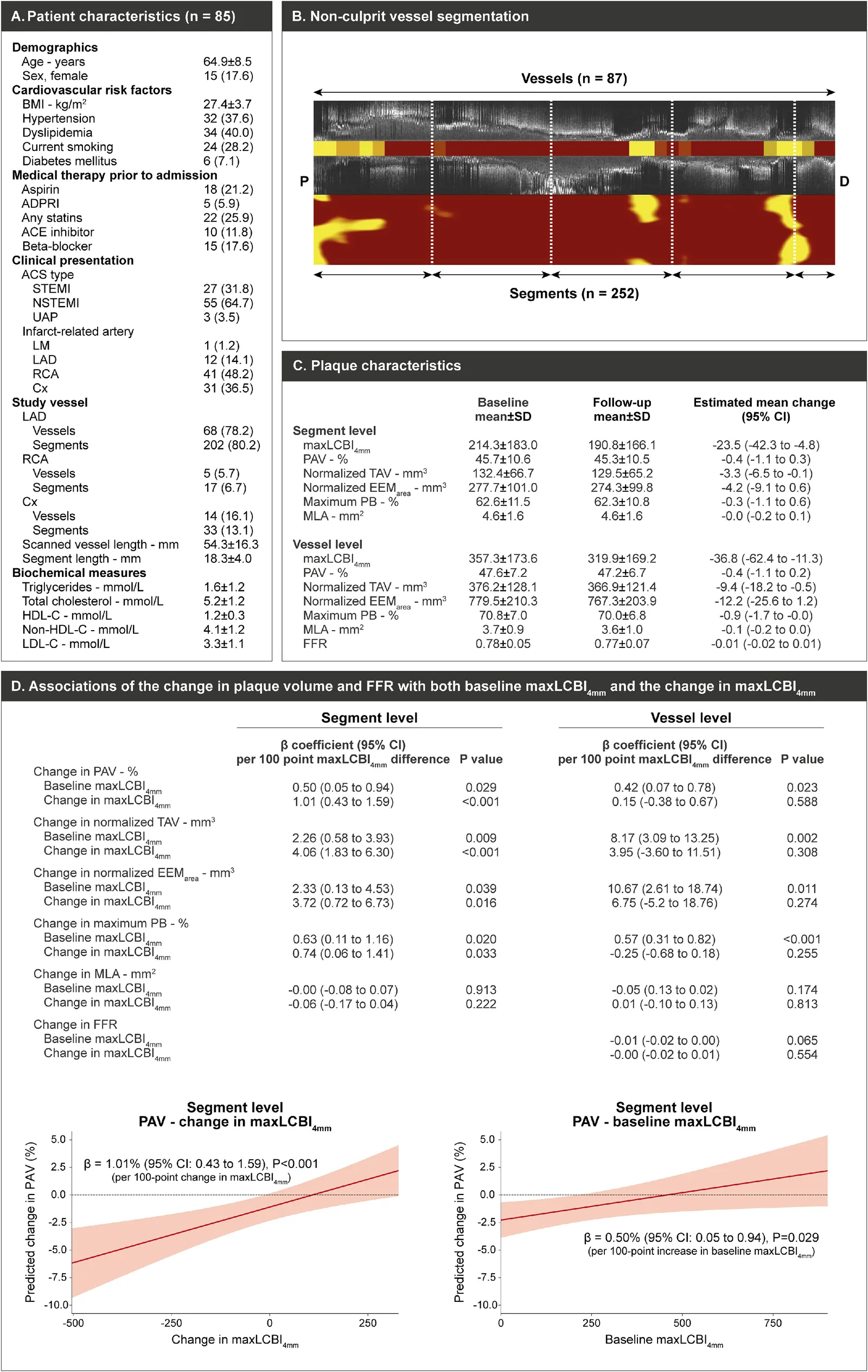
**Panel A.** Baseline characteristics of the patients included in this study. Values are presented as mean±SD or frequency (%). **Panel B.** Representative longitudinal IVUS-NIRS pullback illustrating vessel segmentation. P denotes the proximal end of the artery, and D denotes the distal end. **Panel C.** Segment-level and vessel-level plaque characteristics at baseline, at follow-up, and assessment of the overall changes over time. **Panel D.** Associations of both baseline maxLCBI_4mm_ and the change in maxLCBI_4mm_ with the corresponding changes in atheroma volume parameters. *Abbreviations: ACE, angiotensin-converting enzyme; ACS, acute coronary syndrome; ADPRI, adenosine diphosphate receptor inhibitors (ticagrelor/clopidogrel/prasugrel); BMI, body-mass index; Cx, circumflex artery; EEM_area_, external elastic membrane area; FFR, fractional flow reserve; HDL-C, high-density lipoprotein cholesterol; LAD, left anterior descending artery; LDL-C, low-density lipoprotein cholesterol; LM, left main; maxLCBI_4mm_, maximum lipid core burden index within any 4-mm segment; MLA, minimum lumen area; NSTEMI, non-ST-elevation myocardial infarction; PAV, percentage atheroma volume; PB, plaque burden; RCA, right coronary artery; STEMI, ST-elevation myocardial infarction; TAV, total atheroma volume; UAP unstable angina pectoris.*

### 2.3 Study endpoints

The primary study outcomes consisted of the segment-level associations of both baseline maxLCBI_4mm_ and the change in maxLCBI_4mm_ with the corresponding changes in atheroma volume, for which the change in PAV served as the principal quantitative measure. Additionally, vessel-level changes in atheroma volume and FFR were assessed in relation to the vessel-wide maxLCBI_4mm_.

### 2.4 Statistical analysis

Only vessels and segments with complete parameter assessment were included in the analysis. Changes from baseline to follow-up were estimated using linear mixed-effects models with a patient-level random effect. Segment- and vessel-level associations between coronary lipid burden and changes in plaque volume were assessed using linear mixed-effects models, with both baseline maxLCBI_4mm_ and the change in maxLCBI_4mm_ included as predictors. The models additionally included the baseline plaque volume parameter, randomization group, and achieved low-density lipoprotein cholesterol (LDL-C) level as fixed effects, and patient as a random effect to account for clustering of multiple segments or vessels within individuals. Differences in mean lipid burden between patients with lipid-rich plaque (maxLCBI_4mm_ ≥325) and those without (maxLCBI_4mm_ <325) were assessed using an independent-samples t test. Additionally, subgroup analyses were performed for the segment-level plaque volume parameters using interaction models to assess potential effect modification by treatment group, ACS type, and baseline statin use.

All reported P-values are 2-sided. A P-value <0.05 was considered statistically significant. Statistical analyses were conducted using R-studio version 4.4.2 (R Foundation for Statistical Computing, Vienna, Austria).

## 3 Results

### 3.1 Patient and segment characteristics

A total of 85 patients (mean age 64.9±8.5 years, 17.6% female) with 87 vessels and 252 segments were included in this study (Fig. 1A). The majority of the patients presented with non-ST-elevation myocardial infarction (64.7%) and were statin naïve (74.1%). Overall, mean LDL-C was 3.3±1.1 mmol/L at baseline and decreased to 1.2±0.8 mmol/L at follow-up (mean change, -2.1 mmol/L [95% CI, -2.4 to -1.9]).

### 3.2 Segment-level associations

At baseline, segment-level PAV was 45.7±10.6%, whereas the mean maxLCBI_4mm_ was 214.3±183.0 (Fig. 1C). Both baseline maxLCBI_4mm_ and the change in maxLCBI_4mm_ contributed independently to changes in plaque volume (Fig. 1D). The change in maxLCBI_4mm_ exhibited a graded association with the change in PAV (β = 1.01% per 100-point change [95% CI: 0.43 to 1.59], P<0.001), as well as with normalized TAV, normalized EEM_area_, and maximum PB (Fig. 1D). Thereby, a reduction in maxLCBI_4mm_ was significantly associated with a reduction in plaque volume and reverse plaque remodeling. However, at the same time, higher baseline maxLCBI_4mm_ corresponded to a generally more progressive plaque phenotype, reflected by greater increases in PAV (β = 0.50% per 100-point increase in baseline maxLCBI_4mm_ [95% CI: 0.05 to 0.94], P=0.029), as well as greater increases in normalized TAV, normalized EEM_area_, and maximum PB (Fig. 1D). No significant interactions were observed for treatment group, ACS type, or baseline statin use.

### 3.3 Vessel-level associations

Overall, 47 (54.0%) vessels demonstrated a segment with a maxLCBI_4mm_ ≥325, of which 19 (40.4%) demonstrated multiple segments with a maxLCBI_4mm_ ≥325. The mean maxLCBI_4mm_ across all segments was significantly higher when at least one segment demonstrated a maxLCBI_4mm_ ≥325 (300±101 vs. 125±67, P<0.001), highlighting the presence of increased overall coronary lipid-burden in vessels with a lipid-rich plaque. The segment-level-derived maxLCBI_4mm_ shifted to a different segment within the same vessel in 28 (32.2%) vessels. Unlike the segment-level observations, the change in maxLCBI_4mm_ was not associated with changes in atheroma volume at the vessel level (Fig. 1D). However, consistent with the segment-level findings, higher vessel-level maxLCBI_4mm_ was associated with adverse changes in PAV (β = 0.42% per 100-point increase in baseline maxLCBI_4mm_ [95% CI: 0.07 to 0.78], P=0.023), normalized TAV, normalized EEM_area_, and maximum PB. A trend towards a decrease in FFR (β = -0.01 per 100-point increase in baseline maxLCBI_4mm_ [95% CI: -0.02 to 0.00], P=0.065) was observed (Fig. 1D).

## 4 Discussion

In the present study, we evaluated the interplay between coronary lipid burden and changes in plaque volume after ACS using serial multimodality imaging of relevant non-culprit lesions in high-risk patients treated with high-intensity lipid-lowering therapy during the early post-ACS period. Our findings demonstrate that a reduction in maxLCBI_4mm_ is associated with atheroma regression. However, segments with higher baseline maxLCBI_4mm_ exhibited adverse changes than those with lower values, reinforcing the high-risk nature of lipid-rich plaques reported previously [4]. Consistent associations across all plaque volume measures support the robustness of our findings. On a vessel-level, patients with lipid-rich segments demonstrated increased overall coronary lipid burden, predisposing the vessel toplaque progression and even a trend toward reduced FFR. Together, the present study provides important insights into the challenges and ambiguities associated with lipid-rich lesions.

Our findings should be interpreted in the context of the study design. Only patients with FFR-confirmed relevant non-culprit stenoses were included. Our cohort exhibited markedly higher TAV and maxLCBI_4mm_ compared with post-ACS plaque stabilization studies such as PACMAN-AMI and HUYGENS, potentially reflecting a greater burden of high-risk lesions that may be more prone to plaque progression [5,6]. More importantly, our follow-up was conducted at 12 weeks rather than 1 year, a period during which patients still are in their vulnerable post-event phase [7]. Lipid-rich plaques may remain highly active during this early period, whereas plaque regression may occur after clinical stabilization and prolonged treatment. Our findings may therefore improve the understanding of the residual risk that persists early after ACS despite contemporary antithrombotic, lipid-lowering, and risk-factor-modifying therapies. Intraplaque lipids play a central role in the inflammatory processes underlying atherosclerosis, suggesting that patients with lipid-rich plaques may derive benefit from additional therapeutic strategies such as adjunctive anti-inflammatory treatment [1].

Several limitations need to be addressed. First, this study represents a post-hoc analysis, and its findings should be interpreted with some caution. Second, most coronary artery segments were identified in the left anterior descending artery, limiting the generalizability to the entire coronary tree. Third, imaging pullbacks were divided into 20-mm segments, which may be considered arbitrary. However, we considered 20-mm segments appropriate given the available pullback lengths and the feasibility of deriving segment-level maxLCBI_4mm_, thereby providing an adequate balance between capturing focal plaque biology and minimizing the influence of longitudinal co-registration errors. Finally, segment-level derived maxLCBI_4mm_ shifted to a different segment in a substantial proportion of vessels. Therefore, segment-level changes in maxLCBI_4mm_ likely better reflect local plaque volume changes, whereas vessel-level changes may not capture focal alterations, resulting in attenuated associations.

## Funding

The Radboud University Medical Center received funding by Amgen Europe B.V., Infraredx inc., and Health Holland to execute this study. The funding providers were not involved in study protocol design, data acquisition, or data analysis.

## CRediT authorship contribution statement

**Jonathan Los:** Writing – original draft, Visualization, Methodology, Investigation, Formal analysis, Data curation, Conceptualization. **Frans B. Mensink:** Writing – review & editing, Investigation, Data curation. **Rohit M. Oemrawsingh:** Writing – review & editing, Investigation, Data curation. **Alexander J.J. Ijsselmuiden:** Writing – review & editing, Investigation, Data curation. **Martijn Meuwissen:** Writing – review & editing, Investigation, Data curation. **Jin M. Cheng:** Writing – review & editing, Investigation, Data curation. **Himanshu Rai:** Writing – review & editing, Formal analysis. **Tim J.F. ten Cate:** Writing – review & editing, Investigation, Data curation. **Cyril Camaro:** Writing – review & editing, Investigation, Data curation. **Peter Damman:** Writing – review & editing, Investigation, Data curation. **Lokien X. van Nunen:** Writing – review & editing, Investigation, Data curation. **Aukelien C. Dimitriu-Leen:** Writing – review & editing, Investigation, Data curation. **Marleen H. van Wely:** Writing – review & editing, Investigation, Data curation. **Aysun Cetinyurek-Yavuz:** Writing – review & editing, Formal analysis. **Robert A. Byrne:** Writing – review & editing, Formal analysis. **Niels P. Riksen:** Writing – review & editing, Resources. **Saloua El Messaoudi:** Writing – review & editing, Methodology. **Jan H. Cornel:** Writing – review & editing, Methodology. **Niels van Royen:** Writing – review & editing, Investigation, Data curation. **Robert-Jan M. van Geuns:** Writing – review & editing, Supervision, Resources, Project administration, Funding acquisition.

## Declaration of competing interest

Rohit M. Oemrawsingh reports speaker or consultancy fees from Abbott Vascular, Terumo, and Sahajanand Medical Technologies. Alexander J.J. Ijsselmuiden reports institutional fees from Medtronic, Meril, and Abbott; and consulting fees from Meril, Angiocare, Abbott, Philips, and Translumina. Cyril Camaro reports institutional speaker fees from Astra Zeneca for regional interventional cardiology meetings. Peter Damman reports research grants from Abbott, Philips, Pie Medical Imaging, and Astra Zeneca; and consulting fees from Philips and Abbott. Marleen H. van Wely reports consulting fees from Boston scientific and Abbott vascular. Prof. Niels P. Riksen is financially supported by a consortium grant of the Dutch Heart Foundation (AtheroNeth; 01-001-2024-0601) and a Vici grant of the Netherlands Organization for Health Research and Development (ZonMw; 09150182410037). Prof. Robert A. Byrne reports grants received by the institutions of employment for research or education from Abbott Vascular, Biosensors, Boston Scientific, and Translumina without impact on personal remuneration and does not accept personal payments from medical device or pharmaceutical industry. Prof. Niels van Royen reports research grants from Biotronik, Abbott, Medtronic, and Philips; and speaker fees from Abbott, RainMed, Microport, and Bayer. Prof. Robert-Jan M. van Geuns reports consulting and speaker fees from Abbott Vascular and Astra Zeneca; and institutional research grants from Amgen, InfraRedx, Astra Zeneca, and Sanofi. All other authors report no conflicts of interest.

## Data Availability

The data that support the findings of this study are available from the corresponding author upon reasonable request.

## References

[1] Garcia-Garcia H.M., Sanz-Sanchez J., Pinilla-Echeverri N., Blanco P.J., Bourantas C. (2025). Advances in coronary imaging of atherosclerotic plaques. EuroIntervention.

[2] Mensink F.B., Los J., Reda Morsy M.M., Oemrawsingh R.M., von Birgelen C. (2025). Changes in non-culprit coronary lesions with PCSK9 inhibitors: the randomised, placebo-controlled FITTER trial. EuroIntervention.

[3] Los J., Mensink F.B., Reda Morsy M.M., Nishimiya K., Oemrawsingh R.M. (2026). Short-term lesion-level impact of extensive LDL-C reduction with statins and PCSK9 inhibitors: a pre-specified subgroup analysis of the randomized FITTER trial. Clin Res Cardiol.

[4] Erlinge D., Maehara A., Ben-Yehuda O., Bøtker H.E., Maeng M. (2021). Identification of vulnerable plaques and patients by intracoronary near-infrared spectroscopy and ultrasound (PROSPECT II): a prospective natural history study. The Lancet.

[5] Räber L., Ueki Y., Otsuka T., Losdat S., Häner J.D. (2022). Effect of Alirocumab Added to High-Intensity Statin Therapy on Coronary Atherosclerosis in Patients With Acute Myocardial Infarction: The PACMAN-AMI Randomized Clinical Trial. Jama.

[6] Nicholls S.J., Kataoka Y., Nissen S.E., Prati F., Windecker S. (2022). Effect of Evolocumab on Coronary Plaque Phenotype and Burden in Statin-Treated Patients Following Myocardial Infarction. JACC Cardiovasc Imaging.

[7] Jernberg T., Hasvold P., Henriksson M., Hjelm H., Thuresson M. (2015). Cardiovascular risk in post-myocardial infarction patients: nationwide real world data demonstrate the importance of a long-term perspective. Eur Heart J.

